# Knowledge and Practice of Pelvic Floor Muscle Exercises Among Pregnant Women in Enugu Metropolis, Nigeria

**DOI:** 10.1089/whr.2020.0030

**Published:** 2020-10-08

**Authors:** Humphrey Okeke, Lotachukwu Ifediora, Christian Ogungbe

**Affiliations:** ^1^ Department of Physiotherapy, Faculty of Allied Medical Sciences, University of Calabar, Calabar, Nigeria.; ^2^ Department of Physiotherapy, University of Nigeria Teaching Hospital, Ituku, Nigeria.; ^3^ Department of Physiotherapy, University of Calabar Teaching Hospital, Calabar, Nigeria.

**Keywords:** knowledge, practice, pelvic floor exercise, antenatal, pregnant women

## Abstract

***Background:*** Safe maternity and enhanced neonatal outcomes depend on suitable and comprehensive antenatal program for pregnant mothers. This makes it imperative to adopt strategies aimed at ensuring positive antenatal and postnatal experience for women. To this end, many health care organizations and antenatal clinics teach pregnant women pelvic floor muscle exercises (PFMEs) during their antenatal visits. This study was aimed at assessing the knowledge and actual practice of PFMEs among pregnant women who attend antenatal care in Enugu metropolis. Specifically to assess the level of knowledge of PFMEs among the pregnant women, assess the proportion of the women who practice PFMEs, ascertain whether there exists any difference between knowledge and actual practice of PFMEs, and to identify possible factors that affect compliance to PFMEs.

***Materials and Methods:*** Cross-sectional descriptive design was adopted for this study. Pretested structured questionnaire was administered to the sample of 252 antenatal women in selected antenatal clinics selected through simple random sampling. Data were collected and analyzed using SPSS version 25.

***Results:*** Results show that although majority (71.0%) of the women were taught PFMEs, only 38.37% practice the exercise. The difference in proportion between those who were taught and those who practice PFMEs were statistically significant (*p* < 0.05). Major reasons by the respondents for noncompliance with the exercise routine include forgetting (40.4%), being too tired (35.9%), and being too busy (18.0%).

***Conclusion:*** Despite the relatively high level of knowledge of PFMEs, level of practice was low. Hence, it was recommended that antenatal care providers should explore ways of improving compliance with taught exercise regime such as helping the women identify/develop appropriate cues to exercise.

## Introduction

In most countries of the world the antenatal program is a major component of health promotion and prevention strategies. It is vital for safe maternity and enhanced neonatal outcomes.^1^ To this end, sessions of presentation on antenatal exercises have become a major feature of antenatal talks given to pregnant women during their antenatal visits in some hospitals in our locality. Regular mild to moderate exercises is mainly advocated due to the overall benefits.^2^

A few decades ago, pregnant women in many countries knew little or nothing as regard to the beneficial effects and the safety of mild to moderate exercises both to the pregnant women and the fetuses during pregnancy. However, recent studies have shown that regular practice of such exercises mostly have no risk but is beneficial both for mental and physical health.^2,3^

The pelvic floor muscles are the set of muscles attached to the base of the spine at the back and to the pubic bone in front. These muscles support the bowel, uterus, and the bladder. In addition to supporting the pelvic and abdominal organs, controlling urinary and fecal incontinence, the pelvic floor muscles also play additional role in enhancing sexual functions.^4^ An easy way to identify the action of the pelvic floor muscles is by trying to stop mid-flow when urinating. For women, during the mid-flow hold, they will feel the muscles in the vagina, bladder, and anus tighten and move up. The muscles that tighten and move in the process are the pelvic floor muscles. The pelvic floor muscles, by the nature of their fibers, are capable of both resting tone and squeeze pressure.^5^ It is, therefore, possible that women can maintain some level of pelvic floor muscle strength by some activities of daily living.^6^

However, as women go through pregnancy and childbirth, the potential for injury and weakening of the pelvic floor muscles increases. This often gives rise to the myriad of pelvic floor dysfunction that many experience after childbirth. Among the conditions associated with pelvic floor dysfunction are urinary incontinence (UI), dyspareunia, and other conditions that may be transient or permanent.^7^ These issues can negatively impact on the activities and output of women, and worst of all professional and career women who in some cases may be forced to call in sick at work.

Owing to the level of stress the pelvic floor musculature is often subjected to, particularly in women, it becomes necessary to identify the ideal exercise and use that can help to sustain the level of strength and contractile force needed for efficient functioning of the pelvic floor. The most popular pelvic floor muscle exercise (PFME) is the Kegels exercise and its variants. Kegels exercise was named after an American gynecologist Arnold H. Kegel who first described this form of exercise in 1948.^8^ The Kegel exercise involves repeated contraction and relaxation of the pelvic floor muscles, which is aimed at strengthening the pelvic floor muscles.^9^ To perform Kegel exercise, the bladder has to be empty. The person either sits or lies down and tightens the pelvic floor muscles, holds tight and counts 3–5 seconds.^10^ It can be progressed or modified to form other variants. It is often recommended to be performed multiple times per day for several minutes each time. It needs to be done for at least 1–3 months to begin to have the desired effect. PFMEs for women is designed to strengthen and improve the tone of pelvic floor muscles. Strong pelvic floor muscles help to improve the ability to hold urine and, therefore, help to reduce urinary stress incontinence,^9^ especially after child birth. A large proportion of women experience new onset of UI during pregnancy with an estimated prevalence rate of 35%–67%.^11^ The incidence and prevalence rates for postpartum mothers are also high with estimates of 5%–21% and 15%–45%, respectively.^11^ However, available evidence shows that women who do intensive supervised pelvic floor exercises during pregnancy have reduced risk of postpartum UI.^12^

For pelvic floor muscle training to be effective, certain exercise dosage must be followed. The type of exercise, frequency, intensity, and duration of the training in addition to adherence to the training protocol are of primary importance.^13^ The American Congress of Obstetricians and Gynaecologists^14^ recommends an average of 30 minutes of mild to moderate exercises for most days of the week for pregnant women. This goes to emphasize the point that the effectiveness of PFMEs in achieving strong and healthy pelvic floor requires that the women must not only be taught the exercises, but must be sufficiently sensitized to practice the recommended exercises they are taught at the antenatal clinics.

However, studies have shown that in many cases women do not adhere to the recommended exercise regime.^14–16^ Among the factors identified to affect women's compliance to antenatal exercises is the level of knowledge of the exercise programs.^16,17^ Considering that some studies have argued that gaining knowledge of the PFMEs does not equate to actual practice or performance of the PFMEs routine, this study was designed to assess knowledge and actual practice of PFMEs among pregnant women attending antenatal care in Enugu metropolis. This became more imperative considering that Enugu is a major metropolitan city in southeast Nigeria, with a high turnover of pregnant women. According to federal bureau of statistics,^18^ there was a total of 166,738 birth registrations in Enugu in 2017 alone. Specifically, the study sought to assess the level of knowledge of PFMEs among pregnant women attending antenatal care; assess the proportion of the women who practice PFMEs, to ascertain whether there exists difference between knowledge and actual practice of PFMEs, and to identify possible factors that affect compliance to recommended PFMEs.

The hypothesis tested states that there is no significant difference between knowledge and actual practice of PFMEs.

## Materials and Methods

### Study design

The study design was cross-sectional descriptive design using pretested structured questionnaire administered to a sample of antenatal women.

### Study setting

The study was carried out among antenatal women in Enugu metropolis, which is an urban setting and serves as the capital territory of Enugu state of Nigeria. Enugu state has an estimated population of >3 million.^18^ It is one of the five states of the southeastern geopolitical zone of Nigeria whose language is Igbo. The major occupations within the metropolis are civil service and trading.

### Study population

The study population comprises pregnant women attending antenatal in three hospitals consisting of University of Nigeria Teaching Hospital (UNTH) Ituku Ozala, a federal government-owned hospital; Poly Clinic Asata Enugu, which serves as the state-owned general hospital; and Mother of Christ specialist hospital Ogui Enugu, a mission hospital owned by the Catholic Diocese of Enugu.

### Sampling procedure

Purposive sampling was used to select hospitals where physiotherapists give talk on antenatal and postnatal exercises to pregnant women during antenatal visits. The three hospitals selected had a pooled average patient load of 150 antenatal women per week. Considering that the data collection process lasted 4 weeks, the study population of 600 women (150 × 4) was used for the calculation of sample.

### Sample size

The sample size of 252 was arrived at using Taro Yamani formula,^19^

$$n=N∕\left(1+N{\left(e\right)}^{2}\right),$$

where *n* = the sample size, *N* = population under study, and *e* = margin of error = 0.05.

Hence, applying the aforementioned formula, a sample size of 240 was arrived at. However, to make allowance for nonresponse bias, an additional 5% of the sample was added to make a total sample size of 252 pregnant women for the study. The sample was distributed equally among the three hospitals (84 women per center). Respondents were selected by simple random sampling using a toss of coin at the start whereby head represented even numbers and tail represented odd numbers. Tail fell, and as such, using the attendance register for the antenatal visits, pregnant women having the odd numbers on the register were selected on the clinic day at each center after explaining the exclusion criteria and obtaining informed consent, until the desired sample was attained.

In the three hospitals, the physiotherapists held sessions with the pregnant women two times a week. For the antenatal exercises, the exercise protocols taught include general fitness exercises and PFMEs. The sessions are held in group that usually starts with theoretical presentations and ends with practical demonstrations by the physiotherapist with emphasis on the right stance, positioning, and progression for each exercise regimen. The women were encouraged to attend the antenatal exercise sessions for as many times as possible through their antenatal period.

The focus of this study was on the PFME component. The PFMEs were basically the same in the three centers comprising essentially Kegel's exercises and two auxiliary pelvic floor exercises (“bridging exercises” and “pillow squeeze between thighs,” respectively). Four principal physiotherapists (one per center) and four midwives (one per center) assisted in the administration and collection of the questionnaires.

### Exclusion criteria

The following category of pregnant women were excluded from this study:

1. pregnant women who have not attended antenatal for up to three visits;
2. pregnant women having incompetent cervix and/or pre-eclampsia;
3. women beyond 32 gestational weeks at the time of encounter with researcher; and
4. pregnant women not fluent in either English or Igbo language.

### Study instrument

The study instrument for this study was a self-structured questionnaire comprising three sections with close-ended questions: (1) sociodemographic characteristics of respondents, (2) knowledge profile that assessed the knowledge of PFMEs, and (3) practice profile that assessed the practice of PFMEs by the pregnant women. The questionnaire was validated by expert review in a pretest study. It was self-administered, and was filled and returned at the antenatal clinics during antenatal visits. Igbo (the local language in the area) version of the questionnaire was administered to respondents who were not literate in English language. The reliability of both versions of the questionnaire was assessed by test–retest method. Seven days were observed between test–retest among 40 pregnant women (20 women for each version of the questionnaire) attending antenatal clinic at Enugu state university teaching hospital, Parklane Enugu. All checked items on the questionnaires were summed up at test and retest, and compared. There was agreement percentage ranging from 88% to 98% for the Igbo version and 88.6% to 99.8% for the English version; intraclass coefficient was 0.988 for the Igbo version and 0.992 for the English version; and confidence interval ranges from 0.96 to 0.977 for Igbo version and 0.972 to 0.998 for the English version.

#### Data analysis

Data collected were analyzed using statistical package for social science (SPSS) version 25 to generate the frequency score and percentages of the variables assessed. The result generated were presented in frequency tables and the charts as shown in the following results. Test of hypothesis was done using Pearson moment correlation to test the relationship between the knowledge of PFMEs and the practice of PFMEs. Result was also presented in tabular form (Table 4).

## Results

Table 1 shows the result of the sociodemographic characteristics of respondents. The result shows that majority (51.4%) were aged between 21 and 30 years and the mean age was 30 years, whereas their mean gestational age was 20 weeks (ranging between 12 and 32 weeks), most (76.3%) were of the Christian religion, most (79.2%) were married, majority (57.2%) had up to tertiary level of education, 44.5% were self-employed, majority (51.8%) worked for between 5 and 8 hours, and majority (65.7%) had between 1 and 3 children.

**Table 1.** Frequency Table for Sociodemographic Characteristics

Demographic characteristics	Frequency, N (%)
Age	
11–20	7 (2.9)
21–30	126 (51.4)
31–40	112 (45.7)
Total	245 (100.0)
Religion	
Christianity	187 (76.3)
Islam	58 (23.7)
Total	245 (100.0)
Marital status	
Married	194 (79.2)
Single	46 (18.8)
Divorced	5 (2.0)
Total	245 (100.0)
Level of education	
Secondary	105 (42.8)
Tertiary	140 (57.2)
Total	245 (100.0)
Employment status	
Government employed	109 (44.5)
Self-employed	109 (44.5)
Student	25 (10.2)
Unemployed (applicant)	2 (8.0)
Total	245 (100.0)
Hours of work	
0–4 hours	33 (13.5)
5–8 hours	127 (51.8)
9–13 hours	85 (34.7)
Total	245 (100.0)
No. of children	
1–3	161 (65.7)
4–6	84 (34.3)
Total	245 (100.0)

Table 2 shows the frequency distribution of the knowledge components. Majority (74.3%) said they have heard of PFME. However, not all (71.0%) said they have actually been taught PFME. Among those who said they were taught PFME, all (100%) said they were taught Kegel exercises, pillow squeeze between the thighs, and bridging exercises. On the knowledge of the effects of pelvic floor exercises, 41.6% said it prevents urine leak, 4.5% said it prevents fecal leak, 62.0% said it improves sexual function, and 1.6% said to reduce risk of prolapse pelvic organs.

**Table 2.** Frequency Table of Knowledge of Pelvic Floor Muscle Exercises

Knowledge components	Frequency, N (%)
Heard of PFMEs	
Yes	182 (74.3)
No	63 (25.7)
Total	245 (100.0)
Taught how to do PFMEs	
Yes	174 (71.0)
No	71 (29.0)
Total	245 (100.0)
Type of exercises taught	
Kegel exercises	
Yes	174 (71.0)
No	71 (29.0)
Total	174 (100.0)
Pillow squeeze between the thighs	
Yes	174 (71.0)
No	71 (29.0)
Total	174 (100.0)
Bridging exercises	
Yes	174 (71.0)
No	71 (29.0)
Total	174 (100.0)
Effects of PFMEs	
Prevent urine leak	
Yes	102 (41.6)
No	143 (58.4)
Total	245 (100.0)
Prevent fecal leak	
Yes	11 (4.5)
No	234 (95.5)
Total	245 (100.0)
Enhance sexual function	
Yes	152 (62.0)
No	93 (38.0)
Total	245 (100.0)
Reduce the risk of prolapsed	
Yes	4 (1.6)
No	241 (98.4)
Total	245 (100.0)

PFMEs, pelvic floor muscle exercises.

**FIG. 1.**
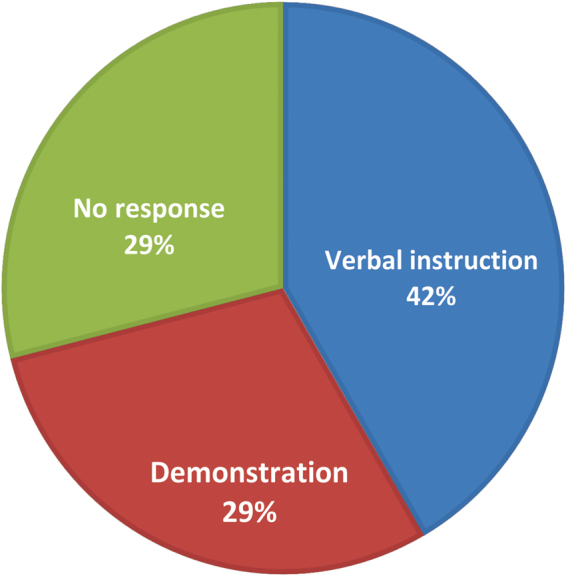
Blue indicates verbal instruction. Green indicates no response. Red indicates demonstration.

Figure 1 shows the distribution of the mode of teaching PFME according to the respondents. Majority (44.1%) said that they were taught by verbal instruction.

Table 3 shows the distribution on the practice of PFMEs. Among those that practice PFME, majority (56.4%) said they practice it two to three times per day.

**Table 3.** Frequency Table for Practice of Pelvic Floor Muscle Exercises

Practice components	Frequency, N (%)
Do you practice PFMEs?	
Yes	94 (38.4)
No	151 (61.6)
Total	245 (100.0)
How often do you do PFMEs per day?	
Once	20 (21.3)
2–3 times	53 (56.4)
>3 times	21 (22.3)
Total	94 (100.0)

**FIG. 2.**
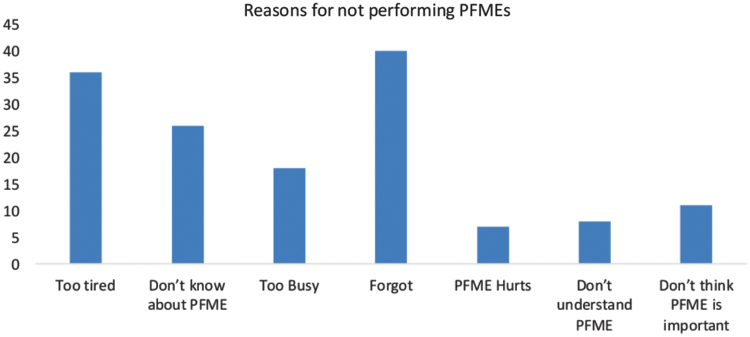
Reasons for not performing pelvic floor muscle exercises. PFMEs, pelvic floor muscle exercises.

Figure 2 shows the distribution of reasons for not performing PFMEs as follows: 40.4% “forgot,” 35.9% “too tired,” 25.7% “did not know about PFMEs,” 18% “too busy,” 11.4% “did not think PFMEs is important,” 8.2% “did not understand PFMEs,” and 7.3% “PFMEs hurt.”

Table 4 shows the result of hypothesis testing. The result shows that although 174 (71.02%) were taught PFMEs, only 94 (38.37%) actually practiced the PFMEs as recommended by the physiotherapist. Test of significance was done using Pearson moment correlation, and the result shows that there was significant difference between knowledge of PFMEs and actual practice (*p* < 0.05).

**Table 4.** Test of Hypothesis Knowledge Versus Practice

Variable	Yes, n (%)	No, n (%)	Total, n (%)	Pearson moment correlation	p
Taught (knowledge of) exercise	174 (71.02)	71 (28.98)	245 (100)		
				0.504	0.000
Practice exercise	94 (38.37)	151 (61.63)	245 (100)		

## Discussion

This study was conducted to study the knowledge and practice of PFMEs among antenatal women in Enugu metropolis with a mean gestational age of 20 weeks (range: 12–32 weeks). As regard to knowledge of PFMEs, it was found that even though 74.8% said they have heard about PFMEs, a slightly lower proportion 71.0% agreed that they were taught how to do PFMEs. The slight disparity in proportion between those who have heard about PFMEs and those who have actually been taught how to perform PFMEs by the physiotherapists indicates that some women probably got information about PFMEs from other sources such as the media (newspapers, television, radio, etc.), family/friends, and even other medical professionals who these women encountered during their antenatal visit other than the physiotherapists. The finding of this study on knowledge of PFMEs contrasts with that of another related study^20^ in which only 37.0% had knowledge of pelvic floor exercises. The plausible reason for the comparatively high percentage of knowledge of PFMEs among the women in this study could be attributed to the weekly sessions on antenatal exercises for pregnant women by physiotherapists at the antenatal clinics in the centers visited. With regard to knowledge of the effect of PFMEs, majority of the women in this study believe that PFMEs prevent urine and fecal leaks, improve sexual function, and reduce risk of prolapse of pelvic organs. These findings are consistent with previous reports.^6,9,12^

Nevertheless, despite the comparatively high proportion on knowledge of PFMEs (71.02%), only 38.37% practice PFMEs. This finding contrasts with the finding in previous studies where the level of knowledge of PFMEs was identified as a key determinant of the level of compliance to PFMEs.^16,17^ Although the knowledge and proper understanding of PFMEs is important for the performance of PFMEs, the findings here reveal that knowledge of PFMEs does not indicate certainty of compliance with practice of PFMEs among pregnant women. Hence, this implies that there may be other factors that could hinder or interfere with the pregnant women's compliance with the recommended exercise regime. In this study, majority of the women (40.4%) said they “forgot” to carry out the exercise, and 35.9% said they were “too tired” to do the exercise. Other reasons given by the women for not performing the PFMEs include “too busy” (18.0%), “did not think PFMEs is important” (11.4%), “did not understand PFMEs” (8.2%), and “PFME hurts” (7.3%). This finding is in agreement with the findings in a similar study,^21^ which concluded that patient-related factors exerted the strongest influence on adherence to pelvic floor exercises in women with UI. The authors in the aforementioned study identified “forgetting to perform the exercise” and “boredom with the exercises” as the factors that most strongly affect low compliance. A related study^22^ identified that in addition to helping the women develop a firm knowledge and understanding of PFMEs, having a regular cue to exercise helped to enhance adherence to exercise. Such cues as “doing the exercise IN the train on the way to work” was identified as very useful in enhancing adherence to PFMEs.^22,23^

## Conclusion

In conclusion, although proper knowledge and understanding of PFMEs is important for the performance and compliance to PFMEs, there exists other patient-related factors that often constitute barriers to the performance of the exercise regime. In this study, such factors as “forgetting,” “tiredness,” and being “too busy” were identified as major barriers to the performance of PFMEs. Hence, it is recommended that the physiotherapists should not stop at teaching the pregnant women PFMEs at the antenatal clinics but should go a step further by helping the women individually to identify proper cues that will enhance their performance and adherence to the exercise regime they are taught at the antenatal clinics. And finally, a study to determine the relationship between the sociodemographic characteristics, knowledge, and the practice of PFMEs is already a focus of future research by the researchers.

### Limitations of the study

The fact that not all the pregnant women in the study area are literate in English language is considered a potential limitation to the study. To reduce the likely bias due to language barrier, the Igbo language version of the questionnaire was adapted. The test–retest carried out reviewed a high reliability score of the instrument. Another potential limitation of the study is the variation in gestational age of the respondents. However, the exclusion of those who have not attended the antenatal classes for up to three times from the study is expected to help reduce the likely bias that may arise based on the variation.

## Abbreviations Used

PFME: pelvic floor muscle exercise
UI: urinary incontinence
